# The spectrum of giant cell arteritis through a rheumatology lens

**DOI:** 10.1038/s41433-024-03153-7

**Published:** 2024-06-19

**Authors:** Muhammad Irfan Khalique, Mousindha Arjunan, Samuel Wood, Sarah L. Mackie

**Affiliations:** 1 Department of Rheumatology, Leeds Teaching Hospitals NHS Trust, Leeds, UK; 2 Department of Ophthalmology, Leeds Teaching Hospitals NHS Trust, Leeds, UK; 3 Leeds Institute of Rheumatic and Musculoskeletal Medicine, University of Leeds, Leeds, UK; 4 NIHR Leeds Biomedical Research Centre, Leeds Teaching Hospitals NHS Trust, Leeds, UK

**Keywords:** Immunological disorders, Autoimmune diseases, Diagnosis

## Abstract

Treatment of giant cell arteritis (GCA) aims initially to prevent acute visual loss, and subsequently to optimise long-term quality of life. Initial prevention of acute visual loss in GCA is well-standardised with high-dose glucocorticoid therapy but in the longer term optimising quality of life requires tailoring of treatment to the individual. The licensing of the IL-6 receptor inhibitor tocilizumab combined with advances in vascular imaging have resulted in many changes to diagnostic and therapeutic practice. Firstly, GCA is a systemic disease that may involve multiple vascular territories and present in diverse ways. Broadening of the “spectrum” of what is called GCA has been crystallised in the 2022 GCA classification criteria. Secondly, the vascular inflammation of GCA frequently co-exists with the extracapsular musculoskeletal inflammation of the related disease, polymyalgia rheumatica (PMR). Thirdly, GCA care must often be delivered across multiple specialities and healthcare organisations requiring effective interprofessional communication. Fourthly, both GCA and PMR may follow a chronic or multiphasic disease course; long-term management must be tailored to the individual patient’s needs. In this article we focus on some areas of current rheumatology practice that ophthalmologists need to be aware of, including comprehensive assessment of extra-ocular symptoms, physical signs and laboratory markers; advanced imaging techniques; and implications for multi-speciality collaboration.

## Abstract (zh)

巨细胞动脉炎(GCA)最初治疗的目的是预防急性视力丧失, 随后为改善远期生活质量。大剂量糖皮质激素可较好地标准化GCA患者急性视力丧失的初步预防, 但改善远期生活质量需要制定个体化治疗方案。IL-6受体抑制剂托珠单抗的批准以及活体血管成像的进步, 改变了GCA的诊疗模式。首先, GCA是一种全身性疾病, 累及多个血管区域并且表型多样。在2022年制定的GCA分类标准中, 拓宽了所谓的GCA的“范围”。其次, GCA的血管炎症经常与相关疾病风湿性多肌痛(PMR)的包膜外肌肉骨骼炎症共存。第三, GCA的护理通常须跨多个专科并且医疗机构需要有效的跨专业沟通。第四, GCA和PMR都可能符合慢性或多相病程；长期管理必须根据每个病人的需要制定个体化治疗方案。本文重点介绍了眼科医生需要了解的当前风湿病临床诊疗领域中的一些知识, 包括对眼外症状、体征和实验室标志物的全面评估；先进的影像诊断技术；以及多学科科协作的重要意义。

A new diagnosis of giant cell arteritis (GCA) is considered a medical emergency, requiring immediate high-dose glucocorticoid therapy to prevent ischaemic visual loss [1]. In the UK, GCA is diagnosed in 2.2 people per 10,000 over-40s annually [2]. The mean age at diagnosis is 73 years, with females 2.6 times more commonly affected than males [2]. Many patients first present to ophthalmology but long-term management is frequently delivered by rheumatologists. Tailoring treatment to the individual patient depends on good clinical assessment of the patient throughout the disease course. In this, close collaboration between ophthalmologists and rheumatologists is essential.

GCA was named by the pathologist Gilmour in 1941, who described a widespread vasculitis with intimal hyperplasia usually accompanied by multinucleated giant cells [3]. Gilmour identified this as the same pathology as those of “temporal arteritis” described by Horton [4]. Nomenclature of the primary systemic vasculitides is based on the portion of the vascular tree predominantly affected [5] and on this basis, GCA is considered a type of “large-vessel vasculitis” [5], although medium-sized vessels may also be involved [6]. Although “temporal arteritis” was considered a rare diagnosis, early European autopsy series revealed that pathological evidence of prior GCA was not so rare in the population: in one Swedish study from the 1960s, evidence of (mostly undiagnosed) prior GCA was found in 1% of 1097 consecutive autopsies [7]. On review of casenotes, some had histories of vague illnesses associated with elevation in erythrocyte sedimentation rate (ESR); the clinical manifestations often seemed to improve or resolve after a few months without specific treatment [7]. Even now, patients may experience GCA symptoms for weeks or months, that may remain undiagnosed until visual symptoms occur and they present acutely to medical care [8].

## Ocular presentations of GCA

Patients with suspected GCA are often referred to ophthalmology if there is visual loss or diplopia, or to rheumatology otherwise [9]. For patients presenting with anterior ischaemic optic neuropathy (AION), the main differential is between arteritic or non-arteritic anterior ischaemic optic neuropathy (A-AION or NA-AION). Although rheumatologists and ophthalmologists have a shared understanding of the basic differences between these two conditions [10] (Table 1), in practice the distinction is not always easy, especially where comorbidities complicate assessment [11, 12]; rheumatologists are often called in to provide an opinion on difficult GCA cases. Communication between the specialities could be enhanced if ophthalmologists were aware that rheumatologists are less familiar with the anatomy of arterial territories (Table 2), the rare complication of ciliary body ischaemia [13, 14], and the various ocular imaging modalities that may be needed to demonstrate choroidal ischaemia in GCA [15]. On optical coherence tomography (OCT), up to 30% of GCA patients presenting with ocular involvement may have hyperreflectivity of the inner nuclear layers, indicating paracentral acute middle maculopathy (PAMM) [16]. Fluorescein angiogram or indocyanine green can be used to help identify delayed or incomplete choroidal filling compatible with GCA [17, 18]. The sensitivity of this finding reduces within a few days of commencing steroid treatment [19]. OCT angiography may be an emerging diagnostic tool to detect ischaemic areas on macula and peripapillary capillaries, but is not able to distinguish between arteritic and non arteritic causes [20]. Cerebral infarcts may also occur in GCA and require co-management with neurology with an awareness of their guidelines [21].

**Table 1:** A summary of basic shared understanding between ophthalmologists and rheumatologists about the differences between arteritic anterior ischaemic optic neuropathy (A-AION) and non-arteritic anterior ischaemic optic neuropathy (NA-AION).

	Arteritic anterior ischaemic optic neuropathy (A-AION)	Non-arteritic anterior ischaemic optic neuropathy (NA-AION)
*Demographics and risk factors*		
Age	Older	Middle years
Other conditions	Polymyalgia rheumatica	Cardiovascular risk factors Anatomical predisposition
*Clinical features*		
Visual loss	Profound	Variable
Disc	Chalky white	Disc at risk in both eyes
Evidence of involvement of other vascular territories	Choroidal ischaemia, headache, jaw claudication, etc.	Rare
*Investigation findings*		
Laboratory markers of inflammation	Elevated	Not elevated
Choroidal perfusion	Delayed or incomplete	Prompt and complete
Temporal artery ultrasound or biopsy	Usually positive	Negative

**Table 2:** Relationship of ischaemic complications in giant cell arteritis to vascular territories supplying the eye, familiar to ophthalmologists but less well-known by many rheumatologists.

Vascular territory	Consequences of interruption of arterial supply	Clinical features
Short posterior ciliary arteries	Arteritic anterior ischaemic neuropathy Choroidal ischaemia Cilioretinal artery occlusion	Profound visual loss; or central, peripheral or altitudinal visual loss Chalky white disc
Central retinal artery	Central (rarely branch) retinal artery occlusion	Significant visual loss Cherry red spot
Pial branches of ophthalmic artery supplying the optic nerve	Posterior ischaemic optic neuropathy	Visual loss with relative afferent pupillary defect, but initially normal fundi
Long posterior ciliary artery supplying sclera, ciliary body	Ciliary body ischaemia +/− involvement of sympathetic and parasympathetic nerves	Variable: ocular hypotony, corneal oedema, anterior uveitis, iris rubeosis; anisocoria
Medial and lateral muscular arteries supplying extraocular muscles	Ischaemia of extraocular muscles	Diplopia (usually transient), with or without clinical features of III, IV, or VI cranial nerve palsy

If GCA is suspected but ocular ischaemic complications have not occurred, the patient is often referred to rheumatology for further management. A recent systematic review and meta-analysis summarised the diagnostic accuracy of various symptoms, signs and laboratory markers in GCA [22]. It is worth characterising the symptoms of GCA clearly at presentation, since when GCA relapses, it often targets the same vessel (usually ipsilateral, sometimes contralateral) that was originally involved.

Rheumatologists often refer to ophthalmology if a patient with suspected or confirmed GCA has new visual symptoms. This is because ischaemic visual symptoms in GCA are considered to warrant higher glucocorticoid doses, discussed further below [9].

## What to look for outside the eye

Recommended aspects of physical examination that can be useful in evaluating patients with suspected GCA are: observing for pallor, sweating or signs of fever; gently palpating the superficial temporal arteries for tenderness, thickening, nodularity or loss of pulsation; looking around the hairline for ulcers (scalp necrosis); looking inside the mouth at tongue and teeth; observing the gait; examining temporomandibular joint, neck (including lymph nodes), shoulders and hips; measuring blood pressure in both arms; and auscultating for vascular bruits. All this should be guided by the clinical history (Table 3).

**Table 3:** A checklist of GCA symptoms to ask about.

Any head pain?	Once the patient has reached the specialist, the presence and nature of headache is a poor diagnostic discriminator [22]. Headache type in GCA has been little-researched and is very variable. The headache of GCA may be in any part of the head, not just the temples; it may be mild or episodic, and in the early stages may respond to caffeine or simple analgesia. It may initially mimic migraine or cluster headache [108] but thunderclap headache would be unusual. It tends to be unlike “normal headache”, so patients may instead call it “head pain” [109]. It is often worse on putting the head on the pillow at night. It may reflect involvement not only of the scalp arteries (e.g. superficial temporal, occipital, posterior auricular arteries) but perhaps also of the middle meningeal artery [110]. Note that many headaches, including migraine, respond to high dose steroids. But failure of headache to respond to steroids is a warning signal to think of other causes than GCA.
Is your head sore to touch?	Hyperalgesia of the scalp is distinct from temporal artery tenderness. It may be localised, often to the crown of the head. It can be tender to brush, comb or style the hair, or even to move the hair around.
Have you noticed anything different about your temples?	Patients may mistake the superficial temporal arteries for veins if they have become pulseless. If a patient or relative volunteers without specific prompting that “their veins have come up” or “lumps appeared on their temples” or that it is tender along the “vein”, this helps direct physical examination.
Does the pain stop you sleeping?	The pain of GCA may profoundly disturb sleep. If present, take extra care with the whole history and any medical advice given, as sleep deprivation can impair patients’ short-term memory.
Any jaw problems? Is it difficult to chew on certain foods, or open your mouth wide? Have had had to change your diet?	Jaw symptoms in GCA are more diverse than the classical description of “jaw claudication” (pain and fatigue in the jaw muscles, beginning shortly after chewing, increasing in severity until chewing stops, and resolving with rest). Jaw claudication is considered one of the most discriminatory symptoms for GCA [22] and a “chewing gum test” has been proposed for use in clinic [111]. Difficulty in opening the mouth wide (trismus) can also occur [112]. This may be described as jaw “stiffness” [27]. The combination of symptoms may cause difficulty eating meat, sandwiches, bagels or bread with a hard crust. Some patients switch to a soup diet.
Any neck pain or stiffness?	Inflammation of the vertebral arteries due to GCA, or cervical interspinous bursitis due to PMR, can cause neck pain. Other neck pathologies can mimic GCA (cervicogenic headache).
Any mouth, tongue, tooth or ear problems?	Tongue claudication might be reported as tingling or pallor in the tongue after prolonged talking or eating. Pain on swallowing, facial pain, toothache, ear pain, deafness or tinnitus, mouth pain or “burning” [113], facial swelling, tongue swelling and cough are all uncommon but possible symptoms of GCA; but they may also be clues to the differential diagnosis. Specialist referral and further investigation is directed by the symptom itself.
Any pain or stiffness around your shoulders or hips? Do your arms or legs get tired or achy when you use them?	See section on polymyalgia rheumatica (PMR) and limb claudication in main text.
Any fever, sweats or weight loss? Have you lost your appetite? Have you felt depressed?	See section on constitutional symptoms in the main text.

When evaluating patients with suspected GCA, it is important to keep a differential diagnosis in mind, and to pursue further investigations if necessary, guided by the clinical features. Until GCA has been confirmed via biopsy or imaging, it is important to avoid premature diagnostic closure, as this can lead to GCA overdiagnosis [23].

*Polymyalgia rheumatica* (PMR) is a disease entity closely related to GCA [24]. Patients often describe stiffness[25] and aches around the shoulders, upper arms, hips, upper thighs and sometimes neck. The stiffness improves with activity, worsens with rest, and improves by the afternoon only to return in the early hours of the morning (“morning stiffness”). PMR stiffness may cause difficulty rising from a chair, getting out of bed or turning over in bed. CRP and ESR are usually raised in a similar pattern to GCA. PMR is usually diagnosed and treated in primary care, but many rheumatologists consider diagnostic boundaries in primary care to be too liberal. In one centre with a rapid-access PMR clinic, rheumatologists evaluating patients referred by primary care with suspected PMR reported that they considered that about 45% of their referrals did not have PMR [26]. Primary care physicians counter that they do not refer clinically-obvious cases of PMR to specialist care because delaying treatment is not desirable.

*Limb claudication* is muscle pain that occurs with exercise and is relieved by rest. In GCA the arms are often affected, sometimes with pallor of the extremities, asymmetry of arm blood pressures, or pulselessness. If there is subclavian steal, arm exercise can also cause dizziness.

*Constitutional (“systemic”) symptoms* occur in both PMR and GCA including malaise, depression, fever, sweats, or weight loss. Weight loss may result from loss of appetite, difficulty with food intake due to GCA-related jaw symptoms [27], or both.

*Aortitis* is common in GCA but may have few specific symptoms or signs, apart from constitutional symptoms; aortitis might sometimes be associated with interscapular or back pain, or cough due to irritation of the recurrent laryngeal nerve.

## Blood tests for GCA

*Laboratory markers of inflammation* (acute phase markers) may be rapidly normalised by glucocorticoid therapy; typically, both ESR and CRP are raised but it is worth taking a blood sample for both markers prior to starting glucocorticoids. In the event of conflicting results, CRP tends to be more reliable [28] as ESR may be elevated by unrelated conditions such as monoclonal gammopathy. However, if CRP is only slightly elevated above the reference range, a clearly-elevated ESR can be a useful clue to inflammatory disease. Where ESR is unavailable, plasma viscosity is a suitable alternative.

*Full (complete) blood count* may identify anaemia and elevation of the platelet count; other findings may include subtle abnormalities of myeloid cell counts that persist despite treatment, possibly revealing a predisposition to the disease [29]. Other components of the *hepatic acute phase response* that may be raised in GCA include serum amyloid A, fibrinogen, haptoglobin, ferritin and alkaline phosphatase [30, 31].

Conflicting statements in the literature on “*GCA with normal inflammatory markers*” are likely explained by different parts of the GCA spectrum captured by different studies, utilising different degrees of diagnostic strictness and different clinical pathways. Hayreh’s report of “occult giant cell arteritis” (defined as patients presenting purely with ocular GCA, proven on temporal artery biopsy, without extra-ocular symptoms or signs) included some patients with a normal ESR, but none with a CRP of less than 5 mg/L [32]. In this and many other well-characterised cohorts, in which a high standard of proof is needed to diagnose GCA, it seems to be rare for GCA to present with a normal CRP before commencing glucocorticoids.

In contrast, in a pooled analysis of 1849 patients from 9 studies, 12.6% of patients diagnosed with GCA had a normal CRP; 17.4% had a normal ESR (3429 patients, 15 studies) [22]. This appears surprisingly high, even considering that some laboratories report CRP of <10 mg/L as “normal” in older people and ESR is sometimes “age corrected”. The source studies were conducted across diverse settings, including rheumatology, and data may have been of variable quality. Overdiagnosis of GCA in some historic cohorts may also contribute [23].

In line with Hayreh’s findings, when we audited our own ultrasound-based diagnostic pathway, it was rare for patients with proven GCA to present with a CRP < 5 mg/L, if they had not been started on glucocorticoids at the time of the blood test [33].

Regardless of the “rule-out” value of normal blood tests, the “rule-in” value of abnormal inflammatory markers is poor [22], given the multiplicity of GCA mimics and high population prevalence of comorbidities that can derange blood tests. Therefore, if GCA is strongly suspected following assessment of symptoms, signs and laboratory markers taken together, most rheumatologists would advise initiating treatment and proceeding to further tests to confirm GCA: vascular imaging, temporal artery biopsy, or both [9].

## Tests to confirm GCA

*Temporal artery biopsy* confirms a pathological diagnosis of GCA; since GCA was originally defined as a pathological construct, a positive biopsy by definition has high face validity as a method for confirming GCA [34]. Pathologists now consider various histological features as consistent with active arteritis [35]. “Healed arteritis” as described by the autopsy studies of Gilmour [3] and Ostberg [7] can sometimes be identified in patients with chronic long-standing disease [36], but the inflammatory infiltrate may persist even after one year of glucocorticoid treatment for GCA [37]. Therefore, contrary to popular teaching, it is rarely “too late” for a temporal artery biopsy.

“Skip lesions” arise because vasculitis of the smaller arteries in GCA often has a multifocal pattern [3] and the biopsy may miss the involved segment of artery. Thus, a negative temporal artery biopsy, although it makes GCA less likely, does not entirely exclude the disease. This can lead to reluctance of clinicians to stop glucocorticoid therapy. In one retrospective study, of 129 temporal artery biopsies, 17 were reported as positive, 10 samples insufficient and 102 biopsies were negative; but only 8 of these 102 had their glucocorticoid therapy stopped immediately [38].

To rheumatologists, a negative temporal artery biopsy still gives valuable information, even though it cannot entirely rule out GCA [39]. It has been estimated that a negative temporal artery biopsy would downgrade the odds that the patient truly has GCA to around 12% of the pre-test odds (i.e., negative likelihood ratio: 0.12) [23]. Nonetheless, absolutely “ruling out” GCA in treated patients can be very difficult. Pragmatic management of “biopsy-negative GCA” may involve rapid glucocorticoid taper with close follow-up as a safety-net in case the biopsy might have missed genuine GCA. In the era before widespread availability of rapid-access vascular imaging for GCA, up to two-thirds of “biopsy-negative GCA cases” may have been overdiagnosed [23]. Updated recommendations on vascular imaging for GCA have recently been published [40] underpinned by a systematic literature review [41] and so imaging is discussed only briefly here.

*Temporal artery ultrasound* was first described by Schmidt in 1995 [42] with the characteristic “halo sign”: a non-compressible, circumferential, hypoechoic abnormality of the temporal artery wall. Less commonly ultrasound may reveal stenosis or occlusion. Sonographer expertise is important, since atherosclerosis and various other diseases can produce similar appearances on ultrasound [43].

Because of false positives and false negatives, ultrasound should be viewed as confirming a clinical diagnosis, not making a diagnosis by itself. Therefore, the result should only be actioned in the context of the overall clinical evaluation, including consideration of differential diagnoses. *Axillary artery ultrasound* may be performed on the same visit as temporal artery ultrasound. The ultrasound findings of axillary artery involvement in GCA include loss of the normal trilaminar appearance of the inner layer of the arterial wall, which becomes thickened and hypoechoic. The “slope sign” (a smooth uniform thickening) [44] distinguishes this appearance from atherosclerosis of the axillary artery which has a craggier and often calcified appearance. Similar appearances may be seen in the subclavian arteries in GCA if visualised. Although axillary artery abnormalities remain detectable despite months of treatment, the temporal artery halo sign may disappear within a few days of high-dose glucocorticoid therapy; therefore, if temporal artery ultrasound is utilised, rapid access is required.

The combined requirement for rapid access and sonographer expertise means that the pathway requires constraints to avoid exceeding capacity (the “too many [negative] ultrasounds” problem [33]). A “gatekeeper” role involving expert clinical assessment may be needed to filter temporal artery ultrasound requests. Although the great temporal artery ultrasound innovators in rheumatology necessarily developed their craft as solo operators, the extraordinary time of the first wave of the COVID-19 pandemic illustrates that it is important to design the pathway to be deliverable via a resilient team, rather than becoming dependent on one individual [45].

Because GCA may spare the temporal artery, a negative temporal artery ultrasound does not entirely rule out GCA. Here, imaging of the aorta and its proximal branches can be a useful complementary test, subject to availability. *Contrast-enhanced* *CT* is widely available but will miss some cases [46]. *Magnetic resonance angiography* can identify oedema of the vascular wall in the acute setting, or dilatation or stenosis during long-term follow-up. *Magnetic resonance imaging (MRI)* of the brain is sometimes useful for identifying infarcts in watershed areas [47] or alternatively may identify alternative explanations for presenting symptoms. It may be combined with *gadolinium-enhanced MRI of the orbits* which has an emerging role in detecting inflammation of retro-orbital structures in GCA [48]. *18-flurodeoxyglucose positron emission tomography combined with low-dose CT (PET-CT)* requires rapid access (similar to the timescale required for temporal artery ultrasound) because high dose glucocorticoid impairs the sensitivity of the test. Rheumatologists may frequently utilise this test during long-term follow-up of GCA patients who develop unexplained elevated inflammatory markers after tapering down to lower glucocorticoid doses (7.5 mg prednisolone daily or less), as it can also yield clues to the appearance of “hidden” non-GCA pathologies such as diverticulitis, abscess, malignancy or occasionally other inflammatory rheumatic diseases.

Rapid access to confirmatory tests is also beneficial because the longer a patient remains on high-dose glucocorticoid therapy, the higher the risk that the diagnostic trail may go cold, and the greater the risk that cognitive biases, including recall bias, may distort diagnostic judgement. Rapid feedback from rapid-access tests can help to hone and maintain diagnostic expertise within the rheumatology team.

## Treating new-onset GCA

Rheumatologists are often involved in making treatment decisions in patients with new-onset GCA. “High-dose” glucocorticoid therapy is needed, but how high exactly? There has never been a clinical trial on this. Some ophthalmology departments use the same starting dose for all GCA patients, but in rheumatology practice it is recognised that most patients with cranial GCA respond symptomatically to 40–60 mg oral prednisolone; a higher dose within this range is generally preferred if there are ischaemic visual manifestations or jaw/tongue claudication [9]. Body weight and comorbidities are also taken into account [9]. All these together are sometimes called patient “phenotype”. For GCA with threatened visual loss, intravenous glucocorticoids are sometimes preferred (see below).

### Relevance of ischaemic visual features

Preventing visual loss, which includes “protecting the second eye” if monocular visual loss has already occurred—is a fundamental tenet of early treatment of GCA. When GCA is strongly suspected, therefore, high-dose glucocorticoid therapy is started as soon as possible. Because of general adherence to this tenet, the counterfactual (what would have happened if glucocorticoids were deferred?) has remarkably little data to inform it, and the literature that exists is easy to misinterpret.

According to a literature review published in 2005 [49], in the era before glucocorticoid therapy was available (before 1950), on average 49.9% of patients with GCA had some degree of vision loss, including partial visual loss, and 17.6% had bilateral blindness; once one eye was involved, 34% of patients developed complete visual loss. After glucocorticoid treatment became available, visual loss was reported in 29.2% and bilateral blindness in 5.8%. Only 3.3% developed visual loss that begun after glucocorticoid had been started [49]. The authors noted higher rates of visual loss in reports from ophthalmology clinics than from rheumatology clinics. GCA patients presenting with a history of transient ischaemic visual symptoms are at higher risk of future visual loss [50]; and GCA patients presenting with unilateral visual loss are at highest risk of progressing to bilateral visual loss [51].

In a report by Liu et al. [52], of 45 patients with GCA presenting with visual symptoms (41 with visual loss) to an ophthalmology centre in Miami, six patients presented with new visual loss (mostly AION) after between 1 month and 6 years of tapering glucocorticoid treatment; the prescribed dose of prednisone at which visual loss occurred was 40 mg daily, 20 mg daily, 10 mg daily, 5 mg alternate days, unknown, and one week after prednisolone cessation. Similarly in a report from two Australian hospitals, of 67 patients with AION due to biopsy-proven GCA, seven had recurrences of ipsilateral AION, between 3 and 36 months later, while taking an average prednisone dose of 16 mg daily [53]. Only one of these seven patients showed “warning signs” such as elevated inflammatory markers or headache in the days or weeks preceding AION recurrence. It is crucial to educate patients to recognise GCA relapse early.

Case series from rheumatology centres in the 1980s and 1990s also include a larger proportion of patients with visual symptoms. For example, in a retrospective review of 239 patients with biopsy-proven GCA from three Spanish rheumatology centres, 28.9% presented with visual symptoms and 14.2% (34 patients) had permanent visual loss in one or both eyes (bilateral in 11 patients) [50]. Of 20 patients who presented with unilateral visual loss and had available data, four progressed to bilateral visual loss: two shortly before, and two shortly (12 h and 24 h) after starting glucocorticoid treatment [50].

Given that ocular symptoms tend to result in presentation to ophthalmology services, it follows that patients in case series will be selected for a bad ocular prognosis if identified from ophthalmology clinics. More generally, a progressive broadening of the spectrum of what is considered to be GCA (setting the “filters” to allow more in) will necessarily lead to an artefactual “improvement” in visual prognosis of the group, akin to the phenomenon of “stage migration” in oncology [54]. This same phenomenon might also partly explains dramatic shifts in the proportion of patients with visual loss after introduction of fast-track GCA clinics.

The observation that patients with prior ischaemic visual symptoms are at greater risk of future visual loss has led to the concept of clinical “phenotypes” of GCA, which can help stratify urgency of initial treatment by diagnostic certainty and presence of ischaemic symptoms. Strongly-suspected cranial GCA with ischaemic (especially visual) symptoms requires immediate treatment without even waiting for blood test results. A more diagnostically-ambiguous case of suspected GCA, in the absence of any ischaemic visual, jaw or tongue symptoms, might be better to defer glucocorticoid treatment until reviewed by a specialist, and/or until blood results are back, and/or until vascular ultrasound can be done the next working day. “GCA phenotypes”, in terms of presence of ischaemia in different vascular territories, does not necessarily imply different molecular pathways, but might be simply anatomical “bad luck” (e.g. inter-individual variations in posterior ciliary artery anatomy). Furthermore, the idea of “phenotypes” should not be taken to indicate stable and unchanging disease subtypes, as the extent of vascular involvement can evolve over the course of the disease.

### How do glucocorticoids work in the acute setting?

The histopathology of GCA is familiar from examination of temporal artery biopsy specimens stained with haematoxylin and eosin. On stained sections, the lumen may appear partially or totally occluded by gross hyperplasia of the intimal layer. It has been pointed out that it appears unlikely that even high-dose glucocorticoids could induce regression of intimal hyperplasia during the first few days of treatment [1], leading to the value of intravenous glucocorticoid therapy being questioned. Small studies reported an association between the degree of intimal hyperplasia and neuro-ophthalmic visual complications of GCA [55], but larger studies failed to replicate this association [56].

After surgical excision, the temporal artery shrinks in size so the appearance of tissue structures on the stained biopsy specimen is not necessarily an exact reflection of how the structure is in vivo. Serial ultrasound studies show that initial oral [57] or intravenous [58] glucocorticoids produce a rapid reduction in size of the temporal artery halo (but little change in the measured dimensions of the intimal-medial complex of the axillary artery), followed by a rebound increase after glucocorticoid cessation [58]. This suggests that, for GCA, the acute action of glucocorticoid is primarily in reducing local inflammatory oedema, thus improving tissue perfusion. This leads to questions about the most appropriate route of administration.

### Route of glucocorticoid administration

For patients with current or recent GCA-related ischaemic symptoms in one eye, usual practice is to give a high a dose of systemic glucocorticoid as is considered safe, based on the reasoning that the non-genomic actions of very high dose glucocorticoids are more rapid in onset than the genomic actions. Such very high doses may be achieved either with high-dose intravenous methylprednisolone or with very high dose oral prednis(ol)one (60 mg daily or more). There has never been a randomised comparison of doses or routes of glucocorticoid in this setting. In practice, the best route of administration depends on whether intravenous glucocorticoids can be arranged without delay, since the hypothetical advantages of intravenous glucocorticoid infusion might be outweighed if there were a delay of many hours before the infusion could be arranged.

For patients with new-onset GCA who do not have ischaemic visual symptoms, more typical of the patients who present directly to rheumatology, there have been two randomised trials comparing intravenous versus oral glucocorticoid therapy. The first trial was an open-label trial that recruited 164 patients [59]. The second trial was double-blinded, but only recruited 27 patients [60]. These trials were designed to test whether choosing intravenous therapy at the start made any difference to disease course over the following 12 or 18 months: relapses or cumulative glucocorticoid dose. Although the smaller trial did suggest a difference between study arms, providing the intravenous glucocorticoid was not counted as part of the cumulative glucocorticoid dose, the larger trial showed no difference in outcomes. Neither trial was powered to show a difference in visual outcomes.

### Questions about biologic therapy in new-onset GCA

In places and circumstances where tocilizumab therapy for GCA may be available (dependent on setting of care and reimbursement routes), rheumatologists are sometimes called upon to advise whether and how biologic therapy should be used alongside glucocorticoid therapy for new-onset GCA. The pivotal trial of the IL-6 receptor inhibitor, tocilizumab, recruited patients who were already in remission on high-dose glucocorticoid therapy and excluded patients with recent intravenous glucocorticoid therapy [61]. Historically it had been proposed from in vitro and animal studies that IL-6 might be pro-angiogenic [62] and neuroprotective [63], and that inhibiting the systemic phase of the disease might come at the cost of exacerbating acute retinal ischaemia. Underlining this concern, a small, single-arm clinical trial, GUSTO, reported on 18 patients treated with three days of intravenous methylprednisolone and an intravenous tocilizumab infusion, followed by one year of weekly tocilizumab therapy, without oral glucocorticoids [64]. One patient developed unilateral AION 15 days after the third glucocorticoid infusion [64].

When tocilizumab is given for new-onset GCA, it is generally given alongside high-dose glucocorticoids particularly if there are already any visual symptoms. There are a few case reports of outcomes of addition of “rescue” subcutaneous tocilizumab therapy alongside continued glucocorticoid therapy in patients with deteriorating visual acuity despite initial high-dose intravenous glucocorticoids. In one small case series, three of five patients had improvement of acuity with “rescue” tocilizumab [65].

Intriguingly a very recent study showed that peripheral blood leucocytes in GCA overproduce reactive oxygen species (impaired redox state) resulting in oxidation of fibrinogen and a pro-thrombotic state; in vitro this is reversed with the addition of tocilizumab [66]. It is too early to tell whether this phenomenon, if it also occurs in vivo, might contribute to the efficacy of tocilizumab in reducing relapse risk and cumulative glucocorticoid dose requirements [61]. The role for antithrombotic and antiplatelet agents in GCA remains uncertain [67].

### Monitoring and mitigating early effects of glucocorticoid toxicity

Rapid infusions of high-dose methylprednisolone may be complicated by cardiac dysrhythmias and so the infusion should be given over at least 30 min. Important short-term toxicities that may occur in the first few days of high-dose glucocorticoid therapy include hypertension, hyperglycaemia, and neuropsychiatric sequelae, including insomnia in almost all patients, anxiety in many, and confusion, psychosis or major depression more rarely [68]. Patients with diabetes should be advised that glucocorticoids can induce hyperglycaemia and a plan put in place for appropriate monitoring. Control of blood glucose levels is particularly important in the context of any immunosuppressive therapy (including glucocorticoids) because uncontrolled hyperglycaemia increases infection risk.

## Long-term management of GCA

### Glucocorticoid dose tapering

Once clinical and laboratory remission has been attained, which may take 4–6 weeks, the glucocorticoid dose is generally tapered. If relapse occurs, the dose is escalated, and once remission is recaptured then the taper restarts from the new dose, perhaps at a slower rate. Patients who relapse repeatedly may never entirely stop glucocorticoids and may need a long-term small “maintenance dose”. In clinical practice, it is important that taper protocols are not rigidly imposed on patients, and the glucocorticoid taper is viewed as a collaborative dose titration to discover the minimum effective glucocorticoid dose for that individual patient.

Before the advent of biologic therapy, it remained customary for many decades for the “default” tapering schema to aim to reduce the dose to zero over 12–18 months provided relapse does not occur [9]. Over recent years, clinical practice for glucocorticoid tapers has been influenced by trials of biologic drugs for GCA [69]. It should be noted that these trials do not necessarily recruit the full spectrum of GCA. The GiACTA trial [61] recruited patients with new-onset or relapsing GCA, who had to have had a historic elevated ESR attributable to GCA supported by either biopsy or imaging, plus active disease within the past 6 weeks, evidenced by signs and symptoms of GCA together with ESR≥30 mm/h, CRP≥10 mg/L, or a positive temporal artery biopsy within 6 weeks prior to baseline. At baseline they had to be taking between 20 and 60 mg prednisolone. They were randomised to 52 weeks of tocilizumab or placebo. Investigators were blinded to CRP results. In this trial [61] participants receiving placebo injections had either a 26-week or a 52-week prednisone taper. At the primary endpoint 52 weeks from randomisation, 9 of 51 (18%) of the participants in the placebo/52-week taper group, and 7 of 50 (14%) of participants in the placebo/26-week taper group, were in sustained remission. By the design of the trial, the median cumulative prednisone dose over 52 weeks was higher in the placebo/52-week taper group (2.608 g) than in the placebo/26-week taper group (1.337 g). Even without tocilizumab, therefore, it might be possible to achieve similar clinical outcomes with a faster taper [61]. The major caveat to this argument is that the 26-week taper was implemented in a trial that avoided recruiting the patients at highest risk (excluded patients: those not in remission at baseline, those with recent iv glucocorticoids, or those with various comorbidities) and provided trial-quality access to expert assessment and advice in the event of relapse. At the time of writing most rheumatologists would be cautious about such a rapid taper if using glucocorticoid monotherapy in standard care.

### Oral immunomodulatory agents

Conventional synthetic disease-modifying antirheumatic drugs (csDMARDs) have historically been utilised as immunomodulatory agents for GCA alongside steroids. However, the only csDMARD with reasonable clinical trial evidence is methotrexate; the other agents have not been adequately tested in clinical trials. A meta-analysis of the methotrexate trials [70] demonstrated that use of methotrexate alongside glucocorticoids reduced relapses, cumulative glucocorticoid dose requirements, and increased the chance of sustained discontinuation of glucocorticoids, without any difference in adverse events between trial arms [70]. Other csDMARDs that have been utilised, but have not been adequately tested for efficacy in clinical trials, include leflunomide [71], azathioprine [72], and mycophenolate [73]. Cyclophosphamide is occasionally used for very severe or refractory cases, based on protocols developed in other types of vasculitis, but toxicity is high [74]. Hydroxychloroquine failed to show benefit in one randomised controlled trial [75]. As none of these agents are licensed for GCA, they must be used off-label. Prescribing of any csDMARD should be initiated by a specialist familiar with their use in this age group, and patients should be monitored for toxicity. In practice, there is wide variation in which patients are prescribed csDMARD, and whether combination therapy with tocilizumab is given. Many rheumatologists reserve csDMARD for patients who have relapsed, or for those with, or at risk of, glucocorticoid toxicity; others prescribe csDMARD at diagnosis as a matter of course for all GCA patients with large-vessel involvement. More data is needed on how best to use csDMARD alongside glucocorticoids in GCA.

### Biologic agents

At the time of writing the only biologic agent licensed for GCA is the IL-6 receptor inhibitor, tocilizumab (TCZ), which is given by weekly subcutaneous injection. The approval for GCA (of the subcutaneous form only) was based on two clinical trials demonstrating reduction in relapse and cumulative glucocorticoid dose [61, 76]. Detailed discussion of the role of TCZ can be found elsewhere [9]. Prescribing in some countries, including the UK National Health Service, is constrained by cost-effectiveness data. The UK National Health Service restricts prescribing of TCZ for GCA to relapsing or refractory cases, and is currently limited to one year, based on a 2018 technology appraisal by the National Institute for Health and Care Excellence [77]. In clinical practice, 1 in 3 patients with GCA relapse during the first year following TCZ cessation, especially those with large-vessel involvement in imaging [78].

As an immunomodulatory agent, TCZ is associated with an increased risk of complications; dose reductions, if required, may be achieved by increasing the interval between injections. As outlined in the product literature, it is contra-indicated by various comorbidities, notably diverticulitis where there is a higher risk of perforation. Animal studies support a role for IL-6 in maintenance and repair of mucosal integrity in the GI tract [79].

Insights into the pathophysiology of GCA from the analysis of temporal artery biopsy specimens has led to the development of various novel therapies and some phase 2 trials have yielded interesting reports. For example the well-documented role of T-cells [80] led to a trial of abatacept [81], and activation of Th17 signalling pathways [80] has led to a trial of secukinumab [82]. In both cases, phase 3 trials are awaited at the time of writing.

When used alongside effective biologic immunomodulatory drugs, glucocorticoid dose may be tapered faster than previously. In a recent report, of TCZ for GCA prednisone was tapered to zero over only 8 weeks, with good outcomes reported [83]. It is unlikely that this would be possible with glucocorticoid monotherapy. A drug that achieves disease control with a lower glucocorticoid dose alongside may be better for patients given that most glucocorticoid-related adverse effects are dose-related.

## The GCA spectrum: history

The concept of “the GCA spectrum” recognises that GCA may present in far more diverse ways and has a more variable prognosis, than traditional teaching might suggest. This shift of the diagnostic boundaries to include a more heterogeneous group of patients, coupled with easier pathways to diagnosis, neatly explains why the incidence of GCA-related visual loss has declined [84] while the number of diagnoses has increased [85]. As articulated by Turnbull [54] these diagnostic shifts and drifts may explain many apparent paradoxes or contradictions in the literature [86].

Just as recruiting from ophthalmology clinics results in enrichment for those with long-term adverse visual outcomes, setting the “filters” wider to capture patients who would formerly have been diagnosed with PMR alone would fundamentally change the case-mix. Unlike Turnbull however we dispute the proposition that PMR is more “benign” than GCA; patients with PMR simply have different needs from patients with GCA. This includes difficulties in disentangling PMR symptoms from non-PMR musculoskeletal pain symptoms. The combination of PMR with GCA carries particular challenges because of the high glucocorticoid doses utilised for GCA.

When Gilmour described the pathology of giant cell arteritis, he identified that aortitis in GCA was widespread and associated with dilatation, whereas inflammation in the branches of the aortic arch and in the carotids was distributed in a focal, bilateral and symmetrical pattern, associated with intimal hyperplasia or even (in the case of the smaller arteries such as the central retinal artery) occlusion [3]. The disease entity “giant cell arteritis” was intended to encompass these common pathological findings often found in one patient simultaneously.

Once effective therapy became available, the main imperative became to “prevent visual loss”. Because not all patients lost vision, and because temporal artery biopsy was the main method of confirming diagnosis, the idea of two clinical “phenotypes” or “disease subsets” [87] of “cranial” and “large-vessel” GCA emerged, that required different diagnostic pathways and perhaps different treatment approaches; it was suggested that patients with isolated “large-vessel” disease might not need such high glucocorticoid doses, as they are at lower risk of visual loss than those with cranial involvement [88].

Whole-body imaging with PET-CT has led to renewed appreciation of Gilmour’s early observation that GCA is often more extensive than symptoms would suggest. The ability to perform serial imaging with ultrasound, coupled with the “cohorting” effect of follow-up in dedicated clinics, has revealed that patients presenting with involvement of only cranial, or only large-vessel, territories may later progress to a “mixed” imaging phenotype involving both types of arterial territory. Since the advent of targeted therapies for GCA, a view of GCA as a single disease entity rather than as two distinct “subsets” is also attractive as it would support using the same therapies for both.

The relationship of PMR to GCA has long been debated amongst rheumatologists. When PMR was first named [89], it was as a disease entity, as opposed to being “the precursor of rheumatoid disease”. PMR was viewed as “distressing yet benign” and could therefore be conveniently and safely treated in primary care [89]. Autopsy studies already showed that some patients diagnosed with PMR also had pathological arteritis, leading to a “polymyalgia arteritica” concept [90] but for clinical diagnosis the clear difference in glucocorticoid requirements kept the diseases separate.

Whilst rheumatologists feared the risk of visual loss in GCA, in the case of PMR they feared that primary care physicians might not diagnose or treat PMR in the same way that they themselves might. As effective treatments for rheumatoid arthritis (RA) began to emerge, a strong focus emerged in rheumatology on risk-mitigation by identifying patients presenting with apparent PMR who actually had “elderly-onset” RA [91] or other serious diseases that might be mimicking PMR [92]. Meanwhile, expertise in clinical diagnosis of PMR developed in primary care. In practice, the recommended exhaustive investigations to rule out “PMR mimics” [93] were seldom performed either in primary care [94] or in secondary care [95], providing the clinical picture appeared convincing and was supported by laboratory markers of inflammation.

For PMR patients who did reach secondary care, there was growing circumstantial evidence from epidemiology that at least some might have a condition related to GCA, to the extent that academic physicians debated whether they were in fact two aspects of the same disease [96]; the focus in GCA on the temporal artery led to reclassification of PMR patients with coexistent temporal arteritis as GCA. Even where no arteritis was evident on biopsy, abnormal cytokine production was noted in PMR arteries [97] suggesting that PMR might be a precursor or “forme fruste” of GCA, with tiny [98] or perivascular [99] foci of inflammation observed in temporal artery biopsies from PMR patients. But also, GCA can be a precursor of PMR: when GCA relapses it quite commonly manifests clinically with PMR symptoms [100].

As Turnbull pointed out, over the same period that concepts of GCA have broadened, concepts of what PMR is have been broadening also. In 1982, Spiera and Davison described 400 patients with “typical PMR”, defined as: over the age of 60, pain and stiffness of shoulder and pelvic girdles, ESR > 60 and clinical response to 10 mg prednisone daily [101]. Of these 400, only four (1%) patients developed clinical features of GCA, and none lost vision. This would currently be considered a very stringent definition of PMR. By 2010, of 176 patients that had presented to us with new-onset PMR and were followed for 5 years, eighteen (10%) were later diagnosed with GCA [102]. This “late GCA” was observed more commonly in patients treated with greater than 15 mg prednisolone daily; we questioned whether treatment of PMR with higher prednisolone doses might be more likely to “mask” or defer the appearance of clinical features of GCA. These PMR patients with higher prednisolone requirements would not have qualified for the strict definition of PMR set by Spiera in 1982. Meanwhile the 2022 GCA classification criteria would capture many patients with what was formerly called PMR [103]. Are the two diseases set to finally merge into one?

Musculoskeletal imaging showed that the characteristic extracapsular pattern of musculoskeletal inflammation of PMR [104] was observed in only a subset of patients with clinically typical PMR. This extracapsular pattern frequently co-exists with the arterial inflammation of GCA [105]. Conversely, imaging frequently reveals vascular inflammation (“subclinical GCA”) in patients with symptoms of clinical PMR only, at least in cohorts recruited from rheumatology clinics [106].

## The GCA spectrum: future prospects

The rheumatology field has recently attempted to reconcile these two apparently conflicting perspectives—the academic concept of a continuous spectrum, combined with the clinical drive to stratify patients according to level of need for care—by creating a new umbrella term of “GCA PMR spectrum diseases” [107]. It is hoped that this will improve granularity and thoroughness of clinical characterisation of patients, and discourage extrapolation of historic evidence from one small part of the spectrum to another part.

## Conclusion

In a world of highly standardised protocols for the treatment of GCA as a “medical emergency” for sound reasons of preventing visual loss, combined with a softening of the boundaries of the GCA diagnosis, Turnbull’s concerns about overdiagnosis and overtreatment have proved prescient.

The message for ophthalmologists is that the disease long familiar to them as “GCA” is only a small part of the broadening spectrum of what rheumatologists now call GCA, at least in the academic literature. Extrapolation of evidence from one part of the GCA spectrum across to the other is increasingly problematic. No longer can we simply aim to assign a simple label of “GCA” after which all treatment decisions become easy. With the “spectrum” concept, rather than “merging” distinct diseases into one, it may become increasingly necessary for ophthalmologists and rheumatologists alike to maximise the granularity of their assessment of patients with the new, broader diagnostic labels, perhaps defining new subgroups based on as yet undetermined soluble or imaging biomarkers. What is certain is that close collaboration between rheumatologists and ophthalmologists will continue to be essential in the management of patients with GCA.
